# Peer Relationships and Depressive Symptoms Among Adolescents: Results From the German BELLA Study

**DOI:** 10.3389/fpsyg.2021.767922

**Published:** 2022-01-03

**Authors:** Adekunle Adedeji, Christiane Otto, Anne Kaman, Franziska Reiss, Janine Devine, Ulrike Ravens-Sieberer

**Affiliations:** ^1^Child Public Health, Clinic for Child and Adolescent Psychiatry, Psychotherapy and Psychosomatics, University Medical Center Hamburg-Eppendorf, Hamburg, Germany; ^2^Faculty of Humanities, North-West University, Mafikeng, South Africa; ^3^Argora Clinic, Psychosomatic Clinic and Outpatient Center, Berlin, Germany

**Keywords:** depression, adolescence, peer relationship, BELLA, mental health

## Abstract

**Background:** Poor mental health affects adolescent development and is associated with health and social outcomes in later life. The current study uses cross-sectional data to explore the understudied aspects of peer relationships as a predictor of depressive symptom severity of adolescents in Germany.

**Method:** Data from the German BELLA study were analyzed. We focused on the most recent measurement point of the BELLA study and analyzed data of 446 adolescents (aged 14–17 years). Peer relationship was measured using four items from the internationally established Patient-Reported Outcome Measurement Information System (PROMIS). Depressive symptoms were assessed via seven items of the German version of the Centre for Epidemiological Studies Short Depression Scale (CES-D). Hierarchical linear regression models were computed to explore the association between depressive symptoms and peer relationships. Hierarchical linear regression models served to determine the added predictive effects of each aspect of peer relationships.

**Result:** The regression model showed that 22% of the variance of the severity of depressive symptoms could be explained by the quality of adolescents’ peer relationships (*F*(1,444) = 125.65, *p* < 0.001). Peer acceptance has the most substantial unique contribution to peer relationship as a predictor of depressive symptom severity (Change in *R*^2^ = 0.05; Change in *F* = 27.01, *p* < 0.001). The gender-specific analysis shows different trends for boys and girls.

**Conclusion:** The quality of peer relationships is a significant predictor of adolescents’ depressive symptoms severity. Improved peer acceptance, dependability, and ease of making new friends are significantly associated with reduced depression symptoms for Germany’s adolescent population.

## Background

The years preceding the transition from adolescence to adulthood are a vulnerable development phase in young peoples’ lives. Adapting coping strategies to deal with difficulties in everyday life facilitates psychosocial and developmental changes (Arnett, 2014). This supposition highlights the importance of an adolescent support system for behavioral development and potential psychopathology (Schulenberg et al., 2004) and its potential link to the adolescent experience of depressive symptoms (Ihle, 2012).

The World Health Organization (WHO) reports one in every six adolescents is diagnosed with a Mood disorder. This makes adolescence one of the most susceptible periods in life to mental health problems (Costello et al., 2005). In a sample of 1,001 adolescents aged 12–17 in Germany, 8.2% reported depressive symptoms (Wartberg et al., 2018). However, this prevalence varies significantly between boys and girls. Female adolescents suffer more often from depressive symptoms (12%) than male adolescents (5%) (Wartberg et al., 2018).

Previous research among children and adolescents has identified links between social factors and depressive symptoms. Lower socioeconomic status (SES) and stressful life situations may contribute to adolescent mental health problems (Reiss et al., 2019). Wentzel and McNamara (1999) suggested that interactions among peers associated with perceived support, serving as a buffer against mental health problems and disorders, including depression. While the quality of interpersonal relationships has been argued to predict well-being and socially competent behavior (Wentzel and McNamara, 1999), the implications of peer relationships for adolescents’ severity of depressive symptoms remain understudied.

### Depressive Symptoms

The current study addresses depressive symptom severity as a subjective measure of discomfort caused by the different depressive symptoms. These symptoms are classified systematically or grouped to measure the nature, pattern and severity of depressive symptoms (National Collaborating Centre for Mental Health (Great Britain) [NCCMH] (ed.), 2010). Previous research exploring adolescents depressive symptoms have identified gender differences in the prevalence, severity and predictor of depression in adolescence (Bennett et al., 2005; Salk et al., 2017; Bulhões et al., 2019). While these studies broadly suggest earlier occurrence and more substantial severity of depressive symptoms in girls, an exploration of these differences indicate that factors such as self-competence may predict the emergence of gender differences in depression (Ohannessian et al., 1999).

### Peer Relationship

Peer relationships, on the other hand, are perceived via acceptance, reliability and intimacy with friends and peers. These broad aspects of peer relationships provide an overview of adolescents’ self-reported quality of relationships and interactions with friends and acquaintances (DeWalt et al., 2013). The current study explores four aspects of peer relationship that explains social roles and sociability and contribute to children and adolescents’ well-being. These aspects are:

(1) Peer acceptance: Conceptualized as the extent to which a child or adolescent is socially accepted by peers (McDonald and Asher, 2018).
(2) Dependability of friends: How well a child or adolescent can count on their friends to provide help and support when needed (Paulsson Do et al., 2020).
(3) Intimacy with friends: Regarded as children and adolescents sense of closeness, attachment, coherence, and self-disclosure with their friends that allows sharing sensitive and personal information (Bauminger et al., 2008).
(4) Easiness to make new friends: This is conceptualized as how easy a child or adolescent can make new friends. It facilitates their connectedness with peers and their social environment (Heinsch et al., 2020).

### Peer Relationship and Adolescents Development

Herman-Stahl and Petersen (1996) found that adolescents dissatisfaction with their social support network is associated with more occurrence of a range of mental health problems. More specifically, Vannatta et al. (2009) concluded that the level of social acceptance–projected by adolescents’ peer popularity–provides a wide range of opportunities for adolescents’ social and psychological development. In terms of intimacy and dependability of friends, the quality of friendships has been described as a critical indicator of adolescent psychosocial adjustment and well-being (Burk and Laursen, 2005). Raboteg-Saric and Sakic (2014) reported that adolescents with a higher quality of friendships showed higher life satisfaction, subjective happiness and self-esteem. Results from a sample of 980 adolescents in Andalusia, Spain, also showed that adolescents who reported greater easiness to make new friends reported higher life satisfaction. Furthermore, difficulty in making friends was associated with more severe depressive symptoms (this association being especially significant among boys) (Gomez-Baya et al., 2019).

Although peer relationships are established as a valid social predictor of adolescents’ psychological functioning and the lack of social support as a predictor of depression, the interconnection between peer acceptance, quality of friendship and easiness to make friends and its association with depressive symptoms remain unclear. Previous studies on the association between peer relationships and depressive symptoms have reported varying results based on the examined aspects of peer relationships and demographic sample characteristics.

Oldenburg and Kems (2016) found in a sample of fifth- and eighth-graders that peer acceptance and friendship are two distinct facets of peer relationships, with differing effects on depressive symptoms. Similarly, in a Portuguese sample of the Health Behavior in School-aged Children (HBSC) study, de Matos et al. (2003) reported that poor peer relationships were associated with stronger depressive symptoms. In contrast, Hazel et al. (2014) suggested a negative association between depressive symptoms and adolescent peer relationships. They argued that efforts to maintain peer relationships might increase stress and consequently increase adolescents’ depressive symptoms.

### The Current Study

The current analysis uses the social capital framework to explore the impacts of social context–constituted by the relationship between individuals–on adolescent mental health outcomes (Ferguson, 2006). Keeping in mind the multidimensional attribute of peer relationships, establishing a clear link between the depressive symptoms and adolescent peer relationship remains crucial to understanding the dynamics and mechanisms for reducing depressive symptoms among Germany’s adolescents.

**FIGURE 1:**
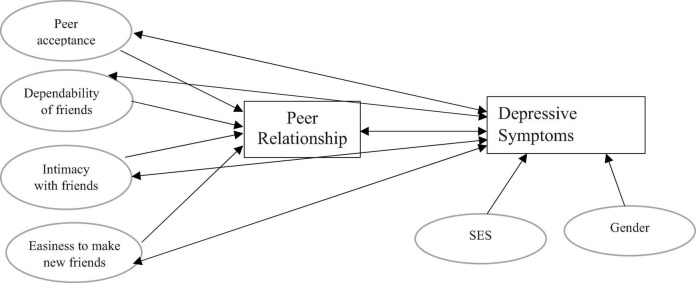
Conceptual framework for the association between aspects of peer relationships and depressive symptoms.

An in-depth understanding of these associations and the significant unique contribution of each aspect of peer relationship to the association, as presented in Figure 1 above, will contribute to the scientific knowledge of the social determinants of depressive symptoms. This will facilitate the development and enhancement of public health interventions to reduce depression in adolescence.

Therefore, the current analysis investigates the association between peer relationships and depressive symptoms among German adolescents. We seek to answer the following question:

1. Do individual aspects of peer relationships and aggregate peer relationships predict adolescent depressive symptoms?
2. Does each aspect of peer relationships has a significant unique contribution to adolescents depressive symptom severity?
3. Is there a significant difference in the association between peer relationships and depressive symptoms severity for boys and girls?

## Materials and Methods

### Study Design and Sample

Data from 446 adolescents aged 14–17 years (*M* = 15.42, SD = 1.14) were analyzed. The subsample was derived from a total sample of 3,492 children, adolescents, and young adults (aged 7–31 years) that participated in the fourth wave of the BELLA cohort study. Only participants aged 14–17 completed the Peer-relationship module of the BELLA study. The BELLA study is the mental health module of Germany’s National Health Interview and Examination Survey for Children and Adolescents (KiGGS). In KiGGS, potential study participants were selected in a random multistage sampling process from the official registers of the residents’ registration offices. The sample comprises 167 sample points throughout Germany. The BELLA study sample is a random subsample of KIGGS. For the fourth wave, data were collected online. Parents provided written informed consent for their 7- to 17-year-old children, and adolescents provided additional consent before completing the questionnaire. The study was approved by the ethics committee of the University Hospital Charité in Berlin and the Federal Commissioner for Data Protection in Germany. Further details on the design and methods of the BELLA Cohort study is available elsewhere (Ravens-Sieberer et al., 2015; Otto et al., 2020).

### Measures

#### Peer Relationships

Peer Relationships were assessed using four items from the Patient-Reported Outcome Measurement Information System (PROMIS)–Pediatric peer relationship measure (DeWalt et al., 2013). The PROMIS peer relationships items were generated based on literature review, expert input, focus groups, and cognitive interviews and then tested in a large group of children (Irwin et al., 2012). These items measure perceived social acceptance and relationship quality and correlate in the expected direction with extensively validated peer-reported outcomes (Devine et al., 2018). Adolescents quality of peer relationships during the past 7 days using a five-point Likert-type scale (ranging from “never” to “almost always”) was captured in four aspects (DeWalt et al., 2013).

(1) Peer acceptance: Liking ratings were collected by asking each participating child and adolescents to rate how much they feel accepted by other kids of their age on a five-point scale from “not at all” to “completely.”
(2) Dependability of friends: The quality of friendship regarding dependability was measured as participants rating of their ability to count on their friends on a five-point scale from “not at all” to “completely.”
(3) Intimacy with friends: Participating adolescents’ subjective evaluation of their closeness with friends was measured as well they can talk about everything with their friends on a five-point scale from “not at all” to “completely.”
(4) Easiness to make new friends: This aspect of peer relationship explains social roles and sociability in children and adolescents. Participants were asked to rate how easy they can make new friends on a five-point scale from “not at all” to “completely.”

In addition to the four aspects of peer relationship, a total peer relationship score was computed as the sum score of the four items ranging from 4 to 20. A higher score implies stronger peer relationships. We observed a Cronbach’s Alpha of 0.81 for the PROMIS peer relationships scale for the current sample.

#### The Severity of Depressive Symptoms

The severity of depressive symptoms was measured using the seven-Item version of the Center for Epidemiological Studies Short Depression Scale [Allgemeine Depressionsskala (ADS), Bailer et al. (2012)]. Participants were asked to rate how often they felt or experienced certain states during the past week. For example, “I was in a happy mood” and “I felt depressed.” Each item was rated using a four-point Likert scale (1 = “rarely or not at all”; 2 = “sometimes”; 3 = “more often”; 4 = “mostly”). An aggregated sum score was computed to generate a total depressive symptoms severity score ranging from 7 to 28. A higher score indicates a high severity of depressive symptoms. A Cronbach’s Alpha of 0.54 was computed for ADS in the current sample.

#### Control Variables

Data on participants’ age in years, gender and SES were collected. The SES was aggregated using the parent-reported revised SES Index of Robert Koch-Institut (2018). This index provides a sum score with values between 3 and 21, reflecting parents’ income, education, and occupation. Household income was calculated by the family’s approximate monthly net income adjusted for household size and age-specific needs, with the total household income score ranging from 1 to 7. Parental education was assessed using the international classification Comparative Analysis of Social Mobility in Industrial Nations (CASMIN) (Brauns et al., 2003). Similarly, the Socio-Economic Index of Occupational Status (ISEI), according to Ganzeboom and Treiman (2003), was used as a criterion for assigning point values to the occupational status. The ISEI index is based on professional activities coded according to the ISCO-08 (International Standard Classification of Occupations) (International Labour Organization [ILO], 2012). These points ranged from 1 to 7 and were generated based on parents’ professional activity and coded according to a standardized procedure on the classification of Professions by the Federal Statistical Office, 2010 (Lampert et al., 2013). The needs-based household net income (net equalized income) was used as an indicator following the requirements of the federal government’s poverty and wealth (Lampert and Kroll, 2009; BMAS - Sozialbericht, 2017).

### Data Analysis

Descriptive statistics were computed for sociodemographic and peer relationship variables. Cronbach’s Alpha was used to evaluate the internal consistency of the analyzed peer relationship scale. Following Nunnally and Bernstein (1994), an acceptable scale should return a minimum alpha of 0.70. Furthermore, the corrected item-total correlations should range between 0.30 and 0.70 in a psychometric sound scale (Ferketich, 1991). A correlation matrix was computed to explore the bivariate associations between aspects of adolescent peer relationships, aggregated peer relationships, the severity of depressive symptoms, SES, and sociodemographic features. Correlation coefficients were interpreted as small (*r* = 0.10), medium (*r* = 0.30), or large (*r* = 0.50) (Cohen, 2013).

Hierarchical linear regression models were computed to test the predictive effect of peer relationships on adolescents’ depressive symptom severity score based on gender and the total sample. An additional hierarchical linear regression model was computed to explore the aspects of peer relationship as predictors of depressive symptom severity. Furthermore, five hierarchical linear regression models were calculated to assess each aspect of peer relationship’s additional/additive predictive value on the severity of depressive symptoms for the total sample and boys and girls. Model 1 explores the added predictive effect of peer acceptance, exploring the relationship between peer relationships and depressive symptoms. Model 2 assesses the unique impact of dependability of friends; model 3 examines the added predictive effect of intimacy with friends, while model 4 explores the added predictive effect of easiness to make new friends. Finally, model 5 presents the impact of all examined aspects of peer relationships on depressive symptoms. Effect sizes and *p*-values are reported for the regression models. The overall fit of the models was evaluated by adjusted *R*^2^ statistics (Nagelkerke, 1991); R-Change and *F*-test determined the significance of changes in model fit. To interpret the regression coefficients of the regression models (β), we used guidelines by Cohen (2013): β = 0.1 indicated a small, β = 0.3 a medium and β = 0.5 a large effect. The significance level was determined as *p* < 0.05 for all analyses. Analyses were computed using IBM SPSS Version 26.

## Results

### Sample Characteristics

Data from adolescents aged 14–17 (*n* = 446) were included in the analysis. As shown in Table 1 below, more than half of the participants were girls (58.7%). 61.2% of participants reported a medium SES.

**TABLE 1:** Sample characteristics (*n* = 446).

Percentage of female		58.7	
Average age		15.8 years	SD = 1.02
Socioeconomic status		**Percentage**	
	High	27.4	
	Medium	61.2	
	Low	11.4	

#### Sample Characteristics–Peer Relationship

**FIGURE 2:**
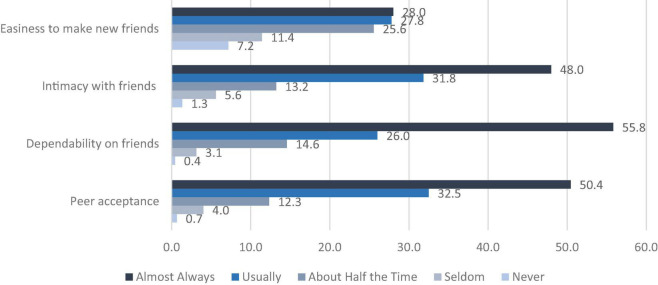
Percentage distribution of aspect of peer relationship.

Descriptive analysis of peer relationships showed that about half of the adolescents felt “almost always” accepted by peers. Consequently, less than 20% reported feeling “never or sometimes” accepted by their peers. Similarly, a little over half (56%) are “almost always” able to count on their friends, and almost half (48%) reported close intimacy with their friends. However, about 45% of the participants reported difficulties making new friends (see Figure 2 below). Analysis of the aggregate peer relationship score returns a score ranging between 4 and 20 and a mean score of 16.4 (SD = 3.17) for the total sample. For boys, an average score of 16.69 (SD = 3.14), while girls reported a mean score of 16.2 (SD = 3.19).

#### Sample Characteristics–Depressive Symptom Severity

The severity score of depressive symptoms ranged between 7 and 28. Descriptive analysis returned a mean score of 11.32 (SD = 3.76) for the total sample, *M* = 10.19 (SD = 3.14) for male adolescents (*n* = 188) and a mean score of 12.09 (SD = 3.96) for female adolescents (*n* = 274).

### Bivariate Analysis

A Pearson product-moment correlation coefficient matrix was computed to examine the associations between aspects of peer relationship, the severity of depressive symptoms, age, and SES score. As presented in Table 2, the aggregated peer relationship score showed a robust negative correlation with the severity of depressive symptoms (*r* = −0.54; *p* ≤ 0.01). Similarly, depressive symptom severity was moderately and negatively associated with each investigated aspect of peer relationships (peer acceptance: *r* = −0.45; *p* ≤ 0.01), dependability on friends (*r* = −0.40; *p* ≤ 0.01), intimacy with friends (*r* = −0.33; *p* ≤ 0.01), and easiness to make new friends (*r* = −0.36; *p* ≤ 0.01). No significant associations were found for age or SES.

**TABLE 2:** Pearson correlation matrix of the severity of depressive symptoms, peer relationship variables, age and socioeconomic status (*n* = 442).

		1	2	3	4	5	6	7	8
1	Severity of depressive symptoms	1	−0.47**	−0.45**	−0.40**	−0.33**	−0.36**	0.04	−0.00
2	Aggregate peer relationship score		1	0.79**	0.84**	0.82**	0.80**	−0.00	0.02
3	Peer acceptance			1	0.56**	0.49**	0.55**	0.03	0.03
4	Dependability on friends				1	0.76**	0.47**	0.02	0.05
5	Intimacy with friends					1	0.45**	−0.01	0.02
6	Easiness to make new friends						1	−0.04	−0.01
7	Age							1	0.00
8	Socioeconomic status score								1

**Correlation is significant at the 0.01 level (2-tailed).

### Regression Model–Peer Relationship and Depressive Symptom Severity

The linear regression model results presented in Table 3 below indicated that peer relationships were significantly associated (adjusted *R*^2^ = 0.22) with the severity of depressive symptoms. Also, separate models assessing the predictive effect of peer relationships on depressive symptom severity return significant results for boys (adjusted *R*^2^ = 0.15) and girls (adjusted *R*^2^ = 0.26). Following Cohen (2013) the predictive effects were stronger for girls (β = −0.51 *p* < 0.001) than for boys (β = −0.39 *p* < 0.001). For the total sample, results from the linear regression model suggest that about 22% (β = −0.47 *p* < 0.001) of the variance in depressive symptom severity is explained by adolescents’ peer relationship quality.

**TABLE 3:** Linear Regression Model: Peer Relationship as a predictor of the severity of depressive symptoms for boys (*n* = 184), girls (*n* = 262), and the total sample (*n* = 446).

	Total			Boys			Girls		
	B	β	*p*	B	β	*p*	B	β	*p*
***Constant***	*20.45*		<*0.001*	*16.46*		<*0.001*	*22.58*		<*0.001*
Peer Relationship	−2.24	−0.47	<0.001	−1.52	−0.39	<0.001	−2.59	−0.51	<0.001
**Model fit Indices**									
Adjusted *R*^2^	**0.22**			**0.15**			**0.26**		
Δ*F*(df1,df2), *p*-value	**Δ*F*(1,444) = 125.65, *p* < 0.001**			**Δ*F*(1,182) = 32.8, *p* < 0.001**			**Δ*F*(1,260) = 92.5, *p* < 0.001**		
**Aspects of Peer relationship**									
***Constant***	*21.2*		<*0.001*	18.27		<0.001	22.94		<0.001
Peer Acceptance	−1.22	−0.28	<0.001	−1.39	−0.36	<0.001	−0.83	−0.19	0.008
Dependability of friends	−0.79	−0.18	<0.001	−0.59	−0.17	0.130	−0.98	−0.21	0.019
Intimacy with friends	0.02	0.01	0.934	0.14	0.05	0.667	−0.32	−0.08	0.370
Easiness to make friends	−0.38	−0.12	<0.05	−0.01	−0.01	0.956	−0.53	−0.16	0.013
**Model fit Indices**									
Adjusted *R*^2^	**0.24**			**0.19**			**0.26**		
Δ*F*(df1,df2), *p*-value	**Δ*F*(4,441) = 35.20, *p* < 0.001**			**Δ*F*(4,179) = 11.67, *p* < 0.001**			**Δ*F*(4,257) = 23.36, *p* < 0.001**		

*Significant values are highlighted in bold. Italic highlights statistical denotation and is not relevant for the interpretation of the results.*

Hierarchical linear regression models (Table 3) show that a complete model that includes all four aspects of peer relationship projects that peer acceptance (β = −0.28 *p* < 0.001), dependability of friends (β = −0.18 *p* < 0.001), and easiness to make new friends (β = −0.12 *p* < 0.05) are all significant predictors of depressive symptom severity. However, intimacy with friends is not a statistically significant unique contributor to the model, even though it is a statistically significant correlate of the dependent variable–depressive symptom severity.

### Regression Model: Added Predictive Effect of Aspects of Peer Relationship and the Severity of Depressive Symptoms

Four hierarchical regression models (models 1–4) were computed to assess the added predictor effects of each of the four aspects of peer relationships and a hierarchical linear regression model (model 5). Model 5 predicts the impact of all four aspects of peer relationships on depressive symptom severity.

As shown in model 1 in Table 4, dependability of friends, closeness with friends and easiness to make new friends were included as predictors of depressive symptom severity. Adding peer acceptance (model 5) showed a more robust added predictive capacity, and an increase in the percentage of variances accounted for by 5%. Similarly, the model exploring the added predictive effect of dependability of friends shows that adding dependability of friends increases the predictive capacity of aspects of peer relationships at predicting depressive symptom severity and increases the percentage of variance accounted for by 1%. However, the model exploring the added effect of closeness to friends (model 3) returns no statistically significant unique contribution to peer relationships. Model 4 examined if making new friends adds anything extra to the prediction of depressive symptom severity, showing that adding this variable increases the predictive capacity by 1%.

**TABLE 4:** Hierarchical regression models exploring the unique contribution of peer relationships to the association between peer relationships and depressive symptoms among German adolescents aged 14–17 years (*n* = 446).

The added effect of peer acceptance	Model 1			Model 2			Model 3			Model 4			Model 5		
	B	β	*p*	B	β	*p*	B	β	*p*	B	β	*p*	B	β	*p*
*Constant*	*19.27*		<*0.001*	20.53		<0.001	21.21		<0.001	21.33		0.000	21.20		<0.001
Peer acceptance				−1.39	−0.32	<0.001	−1.22	−0.28	<0.001	−1.43	−0.33	0.000	−1.22	−0.28	<0.001
Dependability of friends	−0.70	−0.22	<0.001				−0.77	−0.18	0.001	−0.85	−0.20	0.004	−0.79	−0.18	0.008
Intimacy with friends	−1.21	−0.28	0.82	−0.43	−0.11	0.029				−0.05	−0.01	0.834	0.02	0.05	0.934
Easiness to making friends	−0.06	−0.02	<0.001	−0.42	−0.14	0.009	−0.38	−0.12	0.017				−0.38	−0.12	0.017
Adjusted R2	**0.19**			0.23			0.24			**0.23**			0.24		
*F*(df1,df2), *p*-value:	***F*(3,442) = 35.83, *p* < 0.001**			***F*(3,442) = 43.97, *p* < 0.001**			***F*(3,442) = 47.04, *p* < 0.001**			***F*(3,442) = 44.56, *p* < 0.001**			***F*(4,441) = 35.20, *p* < 0.001**		
^(1)^Change in *R*^2^	**0.05**			**0.01**			**0.00**			**0.01**					
^(2)^Change in *F*, *p*-value	**27.01, *p* < 0.001**			**7.09, *p* = 0.008**			0.01, *p* = 0.934			**5.71, *p* = 0.017**					

*Model 1: (1) Change in R^2^ after adding Peer acceptance to model 1 (2) Change in F, and significant of change in F after adding Peer acceptance to model 1.*

*Model 2: (1) Change in R^2^ after adding dependability of friends to model 2 (2) Change in F, and significant of change in F after adding dependability of friends to model 2.*

*Model 3: (1) Change in R^2^ after adding intimacy with friends to model 3 (2) Change in F, and significant of change in F after Intimacy with friends to model 3.*

*Model 4: (1) Change in R^2^ after adding easiness to making friends to model 4 (2) Change in F, and significant of change in F after Easiness to making friends to model 4.*

*Model 5: Evaluate the predictive effect of Peer acceptance, Dependability of friends, Intimacy with friends and easiness to making friends on depressive symptoms. Significant values are highlighted in bold. Italic highlights statistical denotation and is not relevant for the interpretation of the results.*

Generally, peer acceptance, dependability of friends, and easiness to make new friends are all individually unique incremental predictors of the depressive symptom severity. Peer acceptance is the most significant individual contributor to the model (model 5) when modeling the effects of all aspects of peer relationships on depressive symptom severity.

Further analysis of differences in the predictive capacity of the aspects of peer relationship based on gender shows significantly different trends for boys compared to girls. As presented in Table 5, peer acceptance was the sole significant contributor to depressive symptom severity and increased the percentage of variance accounted for by 8%.

**TABLE 5:** Hierarchical regression models exploring the unique contribution of aspects of peer relationships to the association between peer relationships and depressive symptoms among male (*n* = 184) German adolescents aged 14–17 years.

The added effect of peer acceptance	Model 1			Model 2			Model 3			Model 4			Model 5		
	B	β	*p*	B	β	*p*	B	β	*P*	B	β	*P*	B	β	*p*
Constant	15.53		<0.001	17.83		<0.001	18.29		<0.001	18.27		0.000	18.27		<0.001
Peer acceptance				−1.53	−0.40	<0.001	−1.37	−0.36	<0.001	−1.39	−0.36	0.000	−1.39	−0.36	<0.001
Dependability of friends	−0.99	−0.29	0.012				−0.48	−0.14	0.112	−0.59	−0.17	0.119	−0.59	−0.17	0.130
Intimacy with friends	−0.02	−0.01	0.959	−0.17	−0.05	0.520				0.14	0.05	0.669	0.14	0.05	0.667
Easiness to making friends	−0.27	−0.10	0.234	−0.08	−0.03	0.723	0.01	0.00	0.995				−0.01	−0.01	0.956
Adjusted *R*^2^	**0.13**			**0.18**			**0.19**			**0.19**			**0.19**		
Δ*F*(df1,df2), *p*-value:	**Δ*F*(3,180) = 8.75, *p* < 0.001**			**Δ*F*(3,180) = 14.68, *p* < 0.001**			**Δ*F*(3,180) = 15.56, *p* < 0.001**			**Δ*F*(3,180) = 15.64, *p* < 0.001**			**Δ*F*(4,179) = 11.67, *p* < 0.001**		
^(1)^Change in *R*^2^	**0.08**			0.01			0.00			0.00					
^(2)^Change in *F*, *p*-value	**17.95, *p* < 0.001**			2.31, *p* = 0.130			0.19, *p* = 0.667			0.00, *p* = 0.956					

*Model 1: (1) Change in R^2^ after adding Peer acceptance to model 1 (2) Change in F, and significant of change in F after adding Peer acceptance to model 1.*

*Model 2: (1) Change in R^2^ after adding dependability of friends to model 2 (2) Change in F, and significant of change in F after adding dependability of friends to model 2.*

*Model 3: (1) Change in R^2^ after adding intimacy with friends to model 3 (2) Change in F, and significant of change in F after Intimacy with friends to model 3.*

*Model 4: (1) Change in R^2^ after adding easiness to making friends to model 4 (2) Change in F, and significant of change in F after Easiness to making friends to model 4.*

*Model 5: Evaluate the predictive effect of Peer acceptance, Dependability of friends, Intimacy with friends and easiness to making friends on depressive symptoms. Significant values are highlighted in bold.*

For girls, however, three of the four examined aspects of peer relationship, i.e., peer acceptance, dependability on friends, and easiness to make new friends have a significant unique contribution to peer relationship as a predictor of depressive symptoms severity. As shown in Table 6, each of the three aspects increases the variance accounted for by 2%.

**TABLE 6:** Hierarchical regression models assessing the unique contribution of aspects of peer relationships to the association between peer relationships and depressive symptoms among female (*n* = 262) German adolescents aged 14–17 years.

The added effect of peer acceptance	Model 1			Model 2			Model 3			Model 4			Model 5		
	B	β	*p*	B	β	*p*	B	β	*p*	B	β	*p*	B	β	*p*
Constant	21.85		<0.001	22.10		<0.001	22.79		<0.001	23.06		0.000	22.94		<0.001
Peer acceptance				−1.05	−0.24	0.001	−0.85	−0.19	0.007	−1.16	−0.27	0.000	−0.83	−0.19	0.008
Dependability of friends	−1.29	−0.28	0.001				−1.21	−0.26	<0.001	−0.99	−0.21	0.017	−0.97	−0.21	0.019
Intimacy with friends	−0.37	−0.09	0.311	−0.89	−0.21	0.001				−0.44	−0.10	0.225	−0.32	−0.08	0.370
Easiness to making friends	−0.77	−0.24	<0.001	−0.54	−0.17	0.012	−0.56	−0.17	0.009				−0.53	−0.16	0.013
Adjusted *R*^2^	**0.24**			**0.24**			**0.26**			**0.24**			**0.26**		
Δ*F*(df1,df2), *p*-value:	**Δ*F*(3,258) = 28.13, *p* < 0.001**			**Δ*F*(3,258) = 28.78, *p* < 0.001**			**Δ*F*(3,258) = 30.90, *p* < 0.001**			**Δ*F*(3,258) = 28.49, *p* < 0.001**			**Δ*F*(4,257) = 23.36, *p* < 0.001**		
^(1)^Change in *R*^2^	**0.02**			**0.02**			0.00			**0.02**					
^(2)^Change in *F*, *p*-value	**7.09, *p* = 0.008**			**5.58, *p* = 0.019**			0.81, *p* = 0.370			**6.25, *p* = 0.013**					

*Model 1: (1) Change in R^2^ after adding Peer acceptance to model 1 (2) Change in F, and significant of change in F after adding Peer acceptance to model 1.*

*Model 2: (1) Change in R^2^ after adding dependability of friends to model 2 (2) Change in F, and significant of change in F after adding dependability of friends to model 2.*

*Model 3: (1) Change in R^2^ after adding intimacy with friends to model 3 (2) Change in F, and significant of change in F after Intimacy with friends to model 3.*

*Model 4: (1) Change in R^2^ after adding easiness to making friends to model 4 (2) Change in F, and significant of change in F after Easiness to making friends to model 4.*

*Model 5: Evaluate the predictive effect of Peer acceptance, Dependability of friends, Intimacy with friends and easiness to making friends on depressive symptoms. Significant values are highlighted in bold.*

## Discussion

The current study set out to explore the association between peer relationships and adolescents’ depressive symptom severity. It evaluates the added predictive capacity of each of the four examined aspects of peer relationships. The results show that better peer relationship quality significantly corresponds to lower depressive symptom severity for teenage boys and girls. However, for boys, improvement in peer relationships predicts a 15% decline in depressive symptoms. In contrast, an increase in peer relationships for girls corresponds to over a quarter decline in depressive symptom severity. Peer acceptance, dependability of friends, and ease of making friends are significant contributors to peer relationships as predictors of depressive symptoms for the total sample. For boys, peer acceptance was the sole unique statistically significant contributor to peer relationships. For girls, peer acceptance, dependability of friends, and easiness to make new friends all return equal significant individual contributions to peer relationships.

### Socioeconomic and Demographic Predictors of Peer Relationship and Depressive Symptom

The association of adolescent household SES and age with the quality of peer relationships or depressive symptoms severity could not be established for the current sample. The lack of association for age was expected, considering the close age range (14–17). Other studies (Wartberg et al., 2018; Li et al., 2019) exploring depressive symptoms in adolescence with a broader age range have found a positive association between age and depressive symptoms. For example, Wartberg et al. (2018), in a sample of 1,001 adolescents aged 12–17 years in Germany, found that depressive symptoms increase with age. The age distribution of the current sample (age 14–17) centers around middle adolescence when the prevalence of depressive symptom seems stable; this may explain the lack of an association of depressive symptoms and age.

Contrary to findings on the association between the SES and adolescents’ depressive symptoms (Lin et al., 2008), this study found that SES has no significant association with depressive symptoms. On the other hand, Lin et al. (2008) found that adolescents with lower SES reported higher depressive symptoms severity. These contradicting results may be explained by cross-cultural differences. Also, as SES is composed of different variables such as income and education, the interrelation to depressive symptoms may be complex and need further investigation (Goodman and Huang, 2001; Duncan and Magnuson, 2003).

### The Association Between Peer Relationships and Depressive Symptoms Severity

This study shows that the quality of peer relationships is associated with the severity of depressive symptoms for both boys and girls of the studied German sample of adolescents aged 14–17. The results are consistent with prior research and theory proposing that the quality of interpersonal relationships is associated with depressive symptoms and functioning (Burk and Laursen, 2005; Lin et al., 2008; Oppenheimer and Hankin, 2011). The findings suggest that higher peer acceptance, more robust support and deeper intimacy with friends, and easiness to make new friends go in line with lower severity of depressive symptoms.

### Gender Difference in the Association Between Peer Relationships and Depressive Symptoms Severity

Results from the gender-specific analysis show that peer relationship has a more robust predictive capacity for girls’ depressive symptoms than for boys. More specifically, deficit or improvement in peer relationship (peer acceptance, dependability of friends, closeness to friends and easiness to make new friends) have almost double the effect on depressive symptoms for girls (26%) than for boys (15%), i.e., peer relationship seems to have a more substantial buffer effect against depression for girls than for boys or vice versa a lack or low quality of social relationships may affect girls more than boys. The different effect size for boys and girls is consistent with previous studies that have shown discrepancies in the prevalence of depression and the association between quality of peer relationships and depressive symptoms based on gender (Kochel et al., 2017; Krygsman and Vaillancourt, 2017; Van Ryzin and Roseth, 2018).

### The Unique Contribution of Aspect of Peer Relationship to Adolescents Depressive Symptom Severity

Finally, the current study supports the argument that distinctions should be made between the different aspects of peer relationships to truly explore peer relationships’ implication on well-being (Nesi et al., 2018; Steinmayr et al., 2018). While the examination of the correlations between all four aspects of peer relationships and depressive symptoms showed moderate negative associations, further analysis of the added effect of each aspect of peer relationship shows that only three (i.e., peer acceptance, dependability on friends and easiness to make new friends) of the four examined aspects have a uniquely significant contribution to peer relationship as a predictor of depressive symptoms. However, peer acceptance has the most significant individual contribution from those three, accounting for about 5% variance in depressive symptoms. For the total sample, it is assumed that the dependability of friends already covered the effect of intimacy with friends in the model predicting depressive symptoms–that is, having dependable friends is relatively more important than having close friends for adolescents’ depressive symptoms. The gender-specific analysis shows a similar trend for girls with peer acceptance, dependability of friends, and easiness to make new friends, all having an approximately equal unique contribution to peer relationships. However, only peer acceptance has a significant individual contribution to peer relationships accounting for about 8% variance for the boys. These findings set out peer acceptance as an essential predictor of depressive symptoms for German male adolescents. These results are consistent with conclusions from other studies, which show that certain aspects of peer relationships have either little or various effects on depressive symptoms based on gender (Oldenburg and Kems, 2016) and the total sample (Giulietti et al., 2020). Oldenburg and Kems (2016) found in a study among fifth- and eighth-graders that adolescents’ popularity among peers and relationship with friends differed in their association with depressive symptoms between the genders.

### Limitation

Despite several strengths (e.g., examining aspects of peer relationship, quality of analysis data) of this study, future research is needed to address the limitations. First, self-report measures of depressive symptoms can inflate the associations with subjective peer relationships. Future studies exploring objective measures of depressive symptoms in combination with other predictors of psychosocial well-being are necessary to compute the actual predictive effect of peer relationships on depressive symptoms. Similarly, the adopted measure of peer relationship might have ignored other aspects of peer relationship that are crucial for adolescents social and psychological development. Therefore, for future research, a more comprehensive measure of peer relationships is needed to fully understand the patterns and determinants of children and adolescents’ peer relationships. Also, qualitative interview studies may be interesting to explain the empirically found associations in more depth. Second the cross-sectional design of the current study limits the generalizability of the findings. The present analysis included data from adolescents aged between 14 and 17. A more inclusive study covering a broader age range is required to understand the association between peer relationships and adolescents’ depressive symptoms. A longitudinal study that includes adolescents of all ages might help understand the differences and other adolescent or societal features that moderate or mediate the association between peer relationships and depressive symptoms.

## Conclusion

The findings from this study have potential clinical implications for the prevention and treatment of depression among adolescents. The results may reinforce mental health and educational professionals’ awareness of how aspects of peer relationships are associated with depressive symptoms. We suggest supporting peer acceptance and dependability of friends and making new friends in therapeutic, educational, and community settings as a mechanism to reduce depressive symptoms among (German) adolescents. Furthermore, the results support the use of empirically derived interventions (like group therapies) that include social skills components (e.g., D’Amico and Lalonde, 2017; Zolyomi and Schmalz, 2017; Granholm and Harvey, 2018) or treatments that exclusively target interpersonal relationships as beneficial when treating adolescent depressive symptoms. The current study also sheds light on how the association between peer relationship and depressive symptoms differ between boys and girls, most notably how the contributing aspect of peer relationship differs for both genders. Finally, clinical interventions for adolescent depression will benefit from future research that extensively explores other psychosocial variables (like irritability, anxiety, discrimination) that can explain peer relationships and depressive symptoms and investigate mechanisms among several potentially relevant variables in more depth.

## Data Availability Statement

The raw data supporting the conclusions of this article will be made available by the authors, without undue reservation.

## Ethics Statement

The studies involving human participants were reviewed and approved by the Ethics Committee of the University Hospital Charité, Berlin and the Federal Commissioner for Data Protection in Germany. Written informed consent to participate in this study was provided by the participants’ legal guardian/next of kin.

## Author Contributions

All authors contributed to the conception of the research and writing of the current manuscript.

## Conflict of Interest

The authors declare that the research was conducted in the absence of any commercial or financial relationships that could be construed as a potential conflict of interest.

## Publisher’s Note

All claims expressed in this article are solely those of the authors and do not necessarily represent those of their affiliated organizations, or those of the publisher, the editors and the reviewers. Any product that may be evaluated in this article, or claim that may be made by its manufacturer, is not guaranteed or endorsed by the publisher.
